# Antioxidant, Cytotoxic, and Antiproliferative Activities and Total Polyphenol Contents of the Extracts of *Geissospermum reticulatum* Bark

**DOI:** 10.1155/2016/2573580

**Published:** 2016-06-29

**Authors:** Joanna J. Sajkowska-Kozielewicz, Paweł Kozielewicz, Nicholas M. Barnes, Iwona Wawer, Katarzyna Paradowska

**Affiliations:** ^1^Department of Physical Chemistry, Faculty of Pharmacy, Medical University of Warsaw, Banacha 1, 02-097 Warsaw, Poland; ^2^School of Clinical and Experimental Medicine, College of Medical and Dental Sciences, University of Birmingham, Birmingham B15 2TT, UK

## Abstract

*Geissospermum* species are medically important plants due to their health-promoting effects. The objective of this study was to determine the antioxidant ability and antiproliferative and cytotoxic effects of infusions, tinctures, and ethanolic extracts of* Geissospermum reticulatum* barks in relation to the contents of total phenolics and flavonoids. Seven samples of barks were collected in various regions of Peruvian Amazonia. We found that the amount of total phenolics in the studied products varied from 212.40 ± 0.69 to 1253.92 ± 11.20 mg GAE/kg. In our study there is a correlation (*R* ^2^ = 0.7947) between the results of antioxidants assays: FRAP and ORAC for tinctures, infusions, and ethanolic extracts of* G. reticulatum* barks. We have also observed antiproliferative activities of the ethanolic extracts on normal T-cells. These extracts have caused death on malignant cell lines (THP-1 and HL-60) and this data correlates well with their antioxidant capacity measured by ORAC method. Interestingly, the highest concentration of the ethanolic extract was not toxic in the zebrafish embryo developmental assay. Our results indicate that* G. reticulatum* is rich in antioxidants and have cytotoxic and antiproliferative properties. The data suggests potential immunosuppressive role of the extracts. This is the first study presenting the results of chemical and biological analysis of multiple preparations from *G. reticulatum*.

## 1. Introduction

In spite of a lot money being spent on drug discovery, the number of new drugs introduced onto the market is decreasing. One of the reasons can be the rigorous paradigm “one disease, one molecular target, one drug.” However, disease is a complex phenomenon and thus we should be looking for drugs potentially interacting with multiple targets. Plant medicines contain variety of substances and therefore can act via different pathways. Natural medicines are becoming more popular but many studies are needed so that they can become an integral part of pharmacotherapy. Nowadays, nearly half of the world production of medicines is based on natural products. According to estimates, total number of 60,000 plant species have been discovered, 6,000 of them classified but less than 800 surveyed to this date. It is believed that more than 80% of those discovered in the Andes and the Amazon may exhibit medicinal properties [1]. Very interesting are these known for long-term use as components of herbal mixtures or as the infusions of single plant, such as* Geissospermum reticulatum* [2–4],* Uncaria tomentosa* (cat's claw) [1, 5],* Lepidium meyenii* (maca) [1, 6], and* Croton lechleri* (dragon's blood) [1, 7]. Despite their historical and medical importance, there is a lack of data and scientific studies on the profile of substances responsible for the biological properties, including antiradical, anticancer, and anti-inflammatory activity.

*Geissospermum reticulatum* A. Gentry (Apocynaceae) is a tree commonly found in the Amazon [2, 8]. Aqueous infusions from the bark of* Geissospermum* species have been widely used by native people of this region for numerous medicinal purposes. These infusions exhibit antimalarial, antitumoral, antioxidant, nociceptive, and antibacterial activities. There are 12 known species of this genus and five of them have been studied previously (*G. argenteum*,* G. fuscum*,* G. laeve*,* G. reticulatum*, and* G. sericeum*). They are the source of indole alkaloids of various subtypes: strychnan (akuammicine subtype), corynanthean (normacusine B subtype), aspidospermatan (geissovelline subtype), and flavopereirine *β*-carboline [2, 3, 9]. However, detailed information on the constituents and properties of traditional remedies of* Geissospermum* species, especially* G. reticulatum*, has not been reported.

The main aim of our research was to determine antioxidant activity of* G. reticulatum* barks and to perform preliminary* in vitro* research (cytotoxic and antiproliferative properties). What is more, we wanted to establish the content of active compounds present in the plant and determine if a correlation existed between biological activity and chemical properties of traditional remedies of* G. reticulatum* barks.

## 2. Materials and Methods

### 2.1. Plant Materials

Seven samples of dried barks of* Geissospermum reticulatum* were obtained from National Agrarian University-La Molina (Lima, Peru). The barks of* G. reticulatum* were collected (June 2013) in obedience to local regulations in Peru in different regions of Amazon and are stored at National Agrarian University-La Molina (Lima, Peru).

### 2.2. Preparation of Infusions, Tinctures, and Extracts of* G. reticulatum*

Infusions were prepared by adding 100 mL of boiling water to 5 g of cut dry bark and left in the dark for 60 minutes. Tinctures were made by adding 25 mL of 70% ethanol to 5 g of cut dry bark. To prepare ethanolic extracts of* G. reticulatum* barks were powdered and extracted with 60% ethanol (1 : 10 ratio). They were kept in an ultrasonic bath for 15 min before centrifugation at 150 ×g for 30 min. Spin trapping EPR, using PBN (N-*tert*-butyl-*α*-phenylnitrone) and PBN with DMSO, was applied to test for the free radicals production during sonication. No such production was measured (data not shown). Extracts were filtered using Whatman number 1 and stored at 4°C.

### 2.3. Oxygen Radical Absorbance Capacity (ORAC Assay)

The oxygen radical absorbing capacity assay using fluorescein (ORAC-FL) was based on the fact that proposed by Ou et al. [10] with slight modifications by Číž et al. [11]. Briefly, for measurements FL (0.18 mL, final concentration 5.36 × 10^−8^ mol/L) and sample or blank, 0.10 mL, were mixed in a well and incubated at 37°C for 15 minutes, and AAPH (0.20 mL, final concentration 51.51 mmol/L) was added. The fluorescence was measured every 72 s for 90 minutes using an F-7000 Fluorescence Spectrophotometer (Hitachi Europe Ltd., Warsaw, Poland) equipped with a Micro Plate Reader accessory. The excitation wavelength was 485 nm; the emission wavelength was 520 nm. Solutions of AAPH, fluorescein, Trolox, and dilutions of samples were prepared daily in phosphate-buffered saline (PBS, pH 7.4). All experiments were performed in triplicate.

### 2.4. DPPH Scavenging (EPR Test)

The free radical scavenging activity of the extracts was measured* in vitro* by 2,2′-diphenyl-1-picrylhydrazyl (DPPH) assay following the method described earlier [12]. The stock solution was prepared by dissolving 12.5 mg DPPH with 100 mL methanol and stored at 4°C until required, no longer than 12 hours. 50 *μ*L of sample or blank was mixed with 100–500 *μ*L of DPPH. After vortexing the samples were kept for 30 minutes in darkness and then EPR spectra were recorded. The intensity was taken as the double integral of the spectra. Results were expressed as mmol TE/L (mmol Trolox equivalent L^−1^). All experiments were performed in triplicate. EPR measurements were done on a Miniscope MS200 spectrometer (Magnettech GmbH, Berlin, Germany) with parameters as follow: central field 334 mT, sweep range 8 mT, sweep time 30 s, microwave power 10 mW, and modulation amplitude 0.1 mT.

### 2.5. The Ferric Reducing Ability of Plasma (FRAP Assay)

The ferric reducing ability of plasma (FRAP) assay was conducted according to Benzie and Strain protocol [13]. Briefly, 50 *μ*L of sample or 50 *μ*L of freshly prepared FeSO_4_ standard solution was mixed with 1500 *μ*L of working FRAP reagent, and absorbance reading at 593 nm was taken after 4 minutes of thermostatting at 37°C. The working FRAP reagent was prepared by mixing FeCl_3_ and TPTZ (2,4,6-tripyridyl-s-triazine) solutions with acetate buffer (pH 3.6). The results were taken as a mean of three replicates and expressed as millimoles of reduced Fe^3+^ per 100 mL of sample.

### 2.6. Total Phenolic Content

Total polyphenols (TP) were determined following the modified Folin-Ciocalteu colorimetric method. To 20 *μ*L of extract or gallic acid's solution 1580 *μ*L of Millipore water and 100 *μ*L of Folin-Ciocalteu reagent were added. After 5 minutes at room temperature, 300 *μ*L of 20% NaOH was added, and the reaction mixture was thermostated for 20 minutes at 37°C. The absorbance was measured against the blank at 765 nm with Evolution 60S spectrophotometer (Thermo Scientific). Results were expressed as gallic acid equivalents (mg/kg) and expressed as GAE. All experiments were performed in triplicate.

### 2.7. Total Flavonoid Content

Total flavonoids (TF) were determined using the method of Park et al. [14]. In a cuvette, 1.4 mL of Millipore water, 100 *μ*L of extract or catechin's solution, 60 *μ*L of 5% NaNO_2_, and 60 *μ*L of 10% AlCl_3_ were mixed. After 5 minutes of incubation at 25°C 0.4 mL of 1 M NaOH solution was added and absorbance was measured against the blank at 510 nm using Evolution 60S spectrophotometer (Thermo Scientific). Total flavonoid content was calculated as catechin equivalents (mg/g) and expressed as CAE. All experiments were performed in triplicate.

### 2.8. Cell Culture

THP-1 and HL-60 cells were cultured in RPMI-1640 medium (Sigma Aldrich) supplemented with 1% L-glutamine (Life Technologies), 1% penicillin/streptomycin (10,000 units penicillin and 10 mg streptomycin per mL, Sigma Aldrich), and 10% heat-inactivated fetal bovine serum (Sigma Aldrich). Cells were subcultured at a density of 1.5–2 × 10^6^ cells/mL.

### 2.9. Peripheral Blood Mononuclear Cells (PBMCs) Isolation

PBMCs were isolated from whole blood from healthy donors or from Leukocyte Reduced System (LRS) cones using Ficoll-Paque (GE Healthcare). Blood was layered on Ficoll and centrifuged at 900 ×g for 30 minutes. The PBMC layer was mixed with RPMI in a new tube and centrifuged an additional three times, at 400 ×g for 10 minutes, 8 minutes, and 5 minutes, respectively. The study was covered by ethical approval from the University of Birmingham.

### 2.10. Determination of Cell Necrosis Using 7-Aminoactinomycin D

Cells were stimulated for 24 hours and following the incubation were washed with PBS by centrifugation at 400 ×g for 5 minutes. The supernatant was discarded and the cells were resuspended in 7-AAD solution (50 *μ*g/mL, eBioscience) and incubated for 30 minutes at 4°C. Cells were kept on ice and immediately analysed by flow cytometry.

### 2.11. Determination of Cell Proliferation

PBMCs were labelled with eFluor450 proliferation dye (20 *μ*M, eBioscience) before the cells were incubated for 3 days in the absence or presence of the extracts and stimulated with phytohaemagglutinin-L (5 *μ*g/mL, Roche). At the end of the stimulation period the cells were washed twice with PBS and stained with mouse anti-human CD3 antibody PE conjugated (BD Biosciences) for 20 minutes at 4°C. The cells were again washed and resuspended in 0.2% BSA in PBS, before assay by flow cytometry.

### 2.12. The Zebrafish (*Danio rerio*) Embryo Toxicity Test

For the fish studies artificial egg environment (REKO, ISO, 1984) was used. The zebrafish embryo toxicity test was performed according to the published OECD Guidelines [15]. For each experiment 20 fertilized eggs at the beginning of the epiboly stage (4 hours) were used. The selected eggs were exposed to 7 mL of extracts of 0.1 *μ*g/mL* Geissospermum reticulatum* in REKO. The samples were incubated at 28°C for 24 and 48 hours and embryonic development was observed with Leica microscope after 24 h and 48 h. In this study 4-isopropylphenol (20 mg/L) was used as a positive control agent.

## 3. Results and Discussion

### 3.1. Total Phenolics Content

Plant phenolics constitute one of the major groups of compounds acting as primary antioxidants. The amount of total phenolics in the studied products varied from 212.40 ± 0.69 to 1253.92 ± 11.20 mg GAE/kg (Table 1). The highest total polyphenol content was found in ethanolic extract 7, followed by ethanolic extracts 2, 3, and 1. The results for tinctures and infusions follow similar sequence. The total flavonoids contents were also measured (Table 1). The highest contents were observed for ethanolic extracts and tinctures (from 252.42 ± 0.62 to 591.64 ± 1.03 mg CAE/kg). The values for total flavonoids in infusions were significantly lower, varied from 17.16 ± 0.24 to 94.44 ± 0.35 mg CAE/kg. Thus, the selection of solvent is a very important aspect of extraction of flavonoids [16]. The results suggest that flavonoids occurring in* G. reticulatum* barks are better soluble in 60–70% ethanol than in water.

Phenolic compounds are considered to be the major contributor to the antioxidant activity of functional food, fruits, vegetables, and medicinal plants. Our results revealed that barks of* G. reticulatum* have high levels of phenolic compounds and might be potential sources of natural antioxidants even in their most popular serving form, water infusions.

### 3.2. Antioxidant Properties

To measure the antioxidant properties of plant sample, at least two different methods have to be used because antioxidants present in natural products can deactivate radicals by two major mechanisms: single electron transfer (SET) and hydrogen atom transfer (HAT) [17]. The examples of the HAT reaction are DPPH and ORAC assays and the SET reaction is used in FRAP assay. Numerous studies on antioxidants present in plants have been conducted using the DPPH assay and monitoring DPPH radical with a UV spectrometer. However, in the case of color or cloudy samples the use of electron paramagnetic resonance (EPR) spectroscopy is preferred. Antiradical properties of* G. reticulatum* barks assessed using the DPPH-EPR test are presented in Table 2.

A study using the FRAP assay on extracts from 70 medicinal plants, such as* Melissae folium* or* Spiraea herba*, reported that the results were consistent with those obtained by DPPH and ABTS assays [18]. It encouraged us to use this test in the studies of bark extracts. The ethanolic extracts (bark 7: 7.28 mmol/L, bark 3: 7.13 mmol/L) were the most potent in reducing the Fe^3+^-TPTZ complex. It is important to note that tinctures and infusions showed lower antioxidant properties in this assay but the differences were less pronounced than in the other tests.

Table 2 illustrates antioxidant capacity of the studied products. The ethanolic extracts, 7 (86.95 ± 0.58 mmol TE/L), 3 (83.47 ± 0.48 mmol TE/L), 2 (80.57 ± 1.03 mmol TE/L), and 1 (78.30 ± 0.65 mmol TE/L), showed the highest antioxidant capacity values as measured by ORAC assay, followed by the ethanolic extracts 5 and 4 (67.54 ± 0.48 and 64.37 ± 0.23 mmol TE/L, resp.). The results of antioxidant capacity of tinctures were 2–4 times lower than those of ethanolic extracts, and for infusions results were even 2–7 times lower. All results indicate that antioxidant activity of products of* G. reticulatum* barks is high.

**Figure 1:**
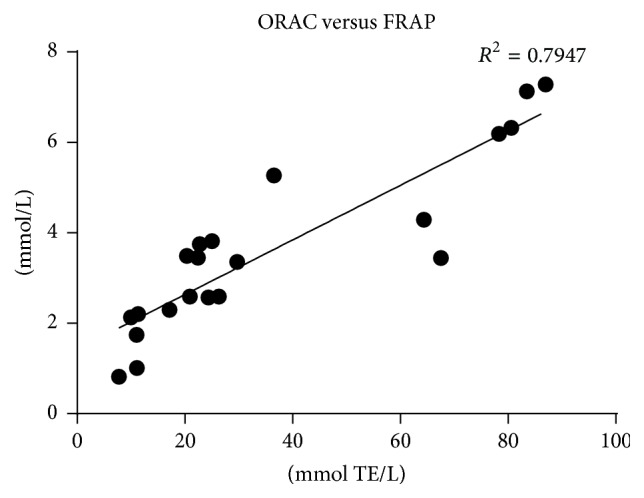
The correlation plot between result of antioxidant assays: ORAC and FRAP.

While there are many publications on the ability of HAT and SET assays to measure the free radical scavenging activity of natural compounds, the results from DPPH, FRAP, and ORAC assays do not always correlate [19, 20]. In our study there is a correlation (*R* ^2^ = 0.7947, Figure 1) between the results of antioxidants assays: FRAP and ORAC for tinctures, infusions, and ethanolic extracts of* G. reticulatum* barks. The presence of such correlation suggests that the compounds present in bark extracts can act as antioxidants by SET and/or HAT mechanisms, at least* in vitro*.

### 3.3. Cytotoxic Activity on* In Vitro* Cultured Cells

**Figure 2:**
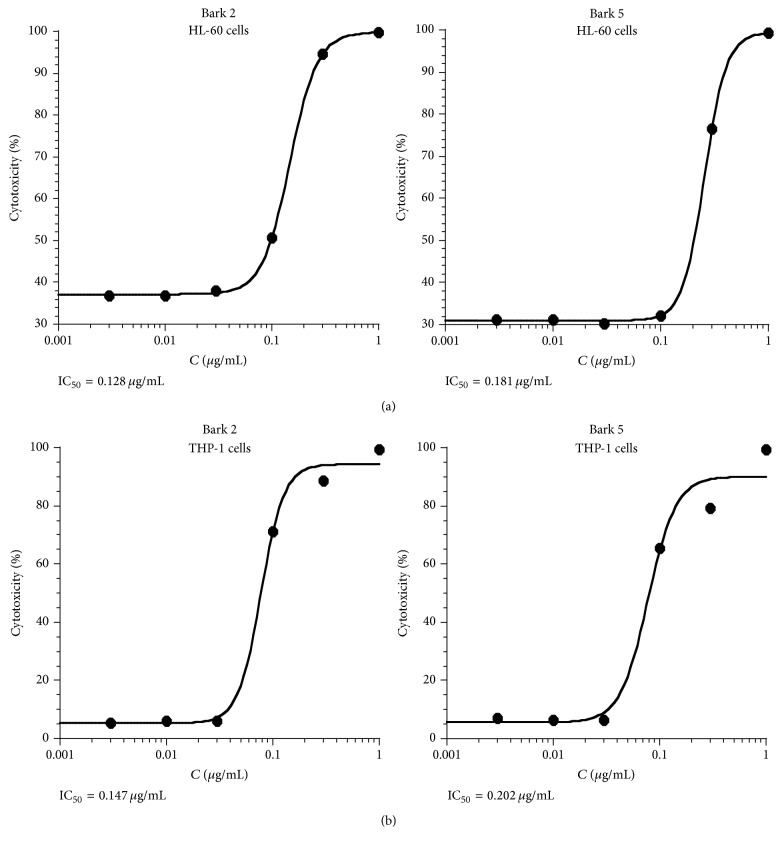
The representative concentration-response plot in (a): HL-60 and (b): THP-1 treated with ethanolic extracts from* G. reticulatum* for 24 hours. (a) Figures show the representative concentration-response plots. The experiments were repeated three times. The data were analysed by iterative curve fitting using a logistic equation and IC_50_ presented as mean. (b) Figures show the representative concentration-response plots. The experiments were repeated twice. The data were analysed by iterative curve fitting using a logistic equation and IC_50_ presented as mean.

**Figure 3:**
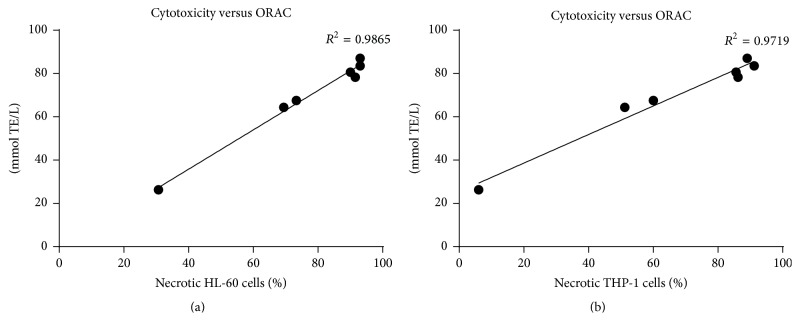
Correlation plots between results of cytotoxicity and antioxidant assays: (a) HL-60 versus ORAC; (b) THP-1 versus ORAC.

The impact of the ethanolic extracts of* Geissospermum reticulatum* upon the viability of the HL-60 and THP-1 cells was assessed after treatment for 24 hours. The concentration-response experiments were performed for the extracts of barks 2 and 5. For the extracts of bark 2 tested on HL-60 and THP-1 the IC_50_ values 0.128 *μ*g/mL and 0.147 *μ*g/mL were obtained, respectively (Figure 2(a)). The bark 5 extracts exhibited more moderate cytotoxicity with IC_50_ 0.181 *μ*g/mL for HL-60 and 0.202 *μ*g/mL for THP-1 (Figure 2(b)). Data from cytotoxicity studies for the extracts at 0.3 *μ*g/mL correlate well with their antioxidant capacity measured by ORAC method (Figure 3).

These results have identified the presence of substances with potential as anticancer agents. Interestingly, the cytotoxicity of the extracts was proportional to their antioxidant capacity, and both of these properties may arise from the presence of the same constituents. Previous studies have shown that tissues in cancer patients produce appreciably higher quantities of reactive oxygen species compared with normal tissue [21]. The possibility that the antioxidants can be at least partly responsible for cytotoxic effect of these extracts should be tested in the future.

### 3.4. Antiproliferative Activity on Human PBMCs

**Figure 4:**
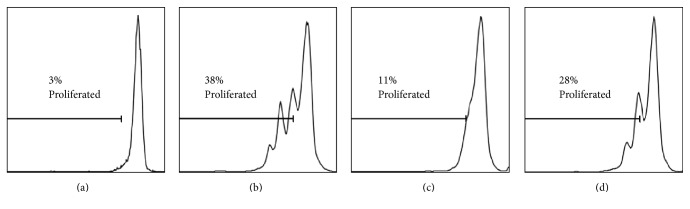
Representative flow cytometry plots: (a) resting; (b) PHA-L; (c) bark 7 [0.1 *μ*g/mL]; (d) bark 7 [0.03 *μ*g/mL].

We also examined the effects of the extracts upon T-cells (CD3+ cells) isolated from PBMCs obtained from healthy donors. T-cells within the PBMC population were induced to proliferate in response to PHA-L (5 *μ*g/mL) [22]. In our experiments 34% of the initial population of anti-CD3 stained cells proliferated after 72-hour stimulation PHA-L (Figure 4).

**Figure 5:**
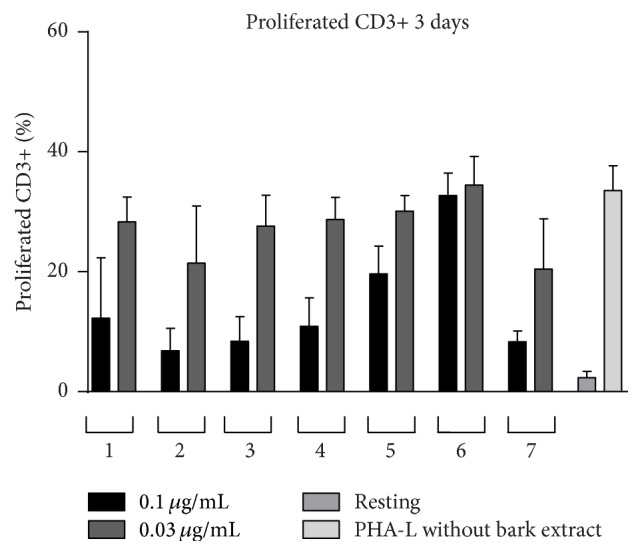
Ethanolic extracts express antiproliferative effect on PHA-L stimulated CD3+ cells (72 hours). Data represented as mean ± SD from 2 independent experiments.

The cells were kept in culture for three days to assess the impact of the extracts at two concentrations (0.1 *μ*g/mL and 0.03 *μ*g/mL). Six out of seven tested extracts showed antiproliferative activity towards PHA-L stimulated anti-CD3 stained PBMCs* in vitro* (Figures 4 and 5). We observed the highest antiproliferative activity for the extracts of barks 2 and 7 (6.8% and 8.3% proliferated cells at 0.1 *μ*g/mL, resp.), whereas the extract from bark 6 at the used concentrations did not show any activity upon the proliferation of human T-cells. The results indicate that chemical compounds present in* G. reticulatum* bark have immunomodulatory properties and potentially can be used in immunosuppressive and anti-inflammatory therapies. The concentrations of the extracts used in this study are relatively low comparing to those used in the other works [3, 23]. Therefore, we could conclude that the bark of* G. reticulatum* may contain a number of very potent molecules, which should be studied in detail. That is why more in-depth investigations have to be performed in order to isolate these compounds in larger amounts and to elucidate the exact mechanism of their antiproliferative action. Now it is too early to forward one.

### 3.5. Effect on Zebrafish

**Figure 6:**
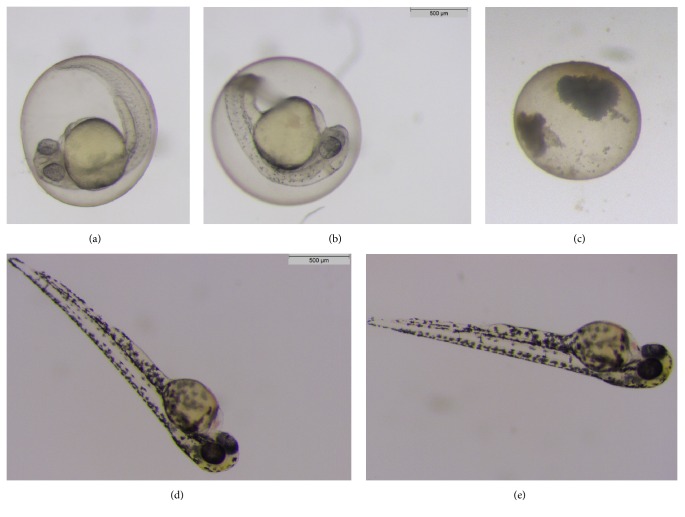
Representative pictures of embryonic development: after 24 hours (a) in REKO, (b) extract 2 [0.1 *μ*g/mL], and (c) 4-isopropylphenol and 48 hours (d) in REKO and (e) extract 2 [0.1 *μ*g/mL].

To test the extracts in* in vivo* model we used the zebrafish embryo developmental assay. Contrary to the results from the* in vitro* tests on malignant cells, the ethanolic extracts did not cause any visible death or deformation of the embryos (Figure 6). The findings of this assay show that the extracts have no teratogenic effects in the doses that are toxic to the cancer cells. There are few possible reasons for this lack of effect. The cytotoxic substances might not be uptaken by zebrafish. The other possibility is the lack of receptors or certain signaling pathways in the zebrafish in which extracts' components are engaged in the* in vitro* cultured cells.

## 4. Conclusions

The* in vitro* antiproliferative and cytotoxic activities of infusions, tinctures, and ethanolic extracts of* Geissospermum reticulatum* barks were evaluated using cancer cell lines and the zebrafish embryo developmental assay. The effects of the extracts were also examined on proliferation of T-cells. The results showed that ethanolic extracts of barks effectively exhibit cytotoxicity upon malignant cultured cells and inhibit proliferation of healthy CD3+ cells. In the search for possible explanation, the antioxidant properties of these materials were studied. The antioxidant activity of bark extracts was directly related to the total amount of phenolics and flavonoids. The good correlation (*R* ^2^ = 0.7947) between the results of antioxidants assays, FRAP and ORAC for tinctures, infusions, and ethanolic extracts of barks, was established. Even simple free radical scavenging assays can be helpful in the fast evaluation of the antioxidant properties of a sample. What is also interesting, the correlation between the results of cytotoxicity and antioxidant ORAC assays was evident: HL-60 versus ORAC (*R* ^2^ = 0.9865) and THP-1 versus ORAC (*R* ^2^ = 0.9719). This may lead to the hypothesis that one of the possible mechanisms of action is linked with free radicals. The data from all assays suggest that the barks of* Geissospermum reticulatum* could serve as free radical inhibitors or scavengers and they are worthy of further study. What is more,* Geissospermum reticulatum* is an interesting source of compounds with immunomodulatory properties and potentially can be used in immunosuppressive and anti-inflammatory therapies. Our studies on the zebrafish model have shown the lack of teratogenicity of the extracts which can indicate they should have neither developmental nor reproductive toxicity.* Geissospermum reticulatum* can be commonly found in the Amazon forest. Taking into account recent popularity of the plants from that region [5, 24, 25] our data further support their potential use as pharmaceutical ingredients. Besides the tested properties, it is highly advantageous for the industry that water and ethanol were used as solvents. It has to be noted that the study deals with crude extracts which are mixtures of various different substances. The follow-up experiments will include fractionations and structural characterizations of these compounds as well as the determination of which particular compounds of the mixture make the most active components of the* G. reticulatum* bark extracts.

## Figures and Tables

**Table 1:** Total phenolic and total flavonoid content.

Sample	Infusions	Tinctures	Extracts
Polyphenol content (mg GAE/kg)			
1	212.40 ± 0.69	553.64 ± 0.54	936.50 ± 8.93
2	559.70 ± 0.87	754.36 ± 0.75	1191.00 ± 4.54
3	353.40 ± 1.37	448.09 ± 1.02	1142.75 ± 9.61
4	416.20 ± 0.98	493.78 ± 1.47	894.00 ± 7.67
5	314.90 ± 0.53	601.64 ± 1.35	788.50 ± 2.11
6	266.70 ± 1.10	443.18 ± 1.57	550.25 ± 1.62
7	545.90 ± 1.14	599.09 ± 1.71	1254.00 ± 11.20

Flavonoid content (mg CAE/kg)			
1	17.16 ± 0.24	471.25 ± 1.46	542.21 ± 0.34
2	84.50 ± 0.50	572.68 ± 1.27	591.64 ± 1.03
3	38.90 ± 0.57	386.02 ± 1.54	419.69 ± 6.24
4	62.83 ± 0.38	410.53 ± 1.19	445.68 ± 6.15
5	52.39 ± 0.36	504.49 ± 1.09	538.58 ± 1.18
6	56.44 ± 0.25	252.42 ± 0.62	274.80 ± 1.55
7	94.44 ± 0.35	434.60 ± 0.66	457.40 ± 2.66

**Table 2:** Antioxidant properties of multiple preparations from *G. reticulatum*.

Sample	Infusions	Tinctures	Extracts
DPPH radical scavenging activity (mg DPPH/L)			
1	0.91 ± 0.02	1.48 ± 0.01	4.36 ± 0.03
2	1.68 ± 0.00	0.90 ± 0.03	5.31 ± 0.04
3	1.36 ± 0.00	0.45 ± 0.01	5.03 ± 0.03
4	1.52 ± 0.01	0.28 ± 0.01	6.79 ± 0.06
5	1.49 ± 0.00	1.63 ± 0.01	4.87 ± 0.11
6	1.12 ± 0.00	0.61 ± 0.01	3.11 ± 0.01
7	1.83 ± 0.00	1.55 ± 0.01	4.64 ± 0.04

FRAP (mmol/L)			
1	1.01 ± 0.01	3.75 ± 0.03	6.28 ± 0.03
2	3.49 ± 0.01	5.27 ± 0.02	6.57 ± 0.05
3	1.74 ± 0.02	2.59 ± 0.01	7.13 ± 0.02
4	2.20 ± 0.02	2.57 ± 0.00	5.16 ± 0.07
5	2.13 ± 0.03	3.82 ± 0.01	4.27 ± 0.06
6	0.82 ± 0.01	2.30 ± 0.01	3.86 ± 0.03
7	3.45 ± 0.01	3.36 ± 0.02	7.28 ± 0.01

ORAC (mmol TE/L)			
1	11.08 ± 0.22	22.75 ± 0.50	78.30 ± 0.65
2	20.34 ± 0.18	36.53 ± 0.22	80.57 ± 1.03
3	11.05 ± 0.13	20.91 ± 0.57	83.47 ± 0.48
4	11.29 ± 0.26	24.34 ± 0.37	64.37 ± 0.23
5	9.98 ± 0.14	25.03 ± 0.09	67.54 ± 0.48
6	7.76 ± 0.10	17.16 ± 0.58	26.30 ± 0.49
7	22.40 ± 0.77	29.67 ± 3.13	86.95 ± 0.58
